# Physical Time Within Human Time

**DOI:** 10.3389/fpsyg.2022.718505

**Published:** 2022-03-30

**Authors:** Ronald P. Gruber, Richard A. Block, Carlos Montemayor

**Affiliations:** ^1^Stanford University Medical Center, Stanford University, Stanford, CA, United States; ^2^Department of Psychology, Montana State University, Bozeman, MT, United States; ^3^Department of Philosophy, San Francisco State University, San Francisco, CA, United States

**Keywords:** passage, IGUS, enduring-self, dualistic, temporality, persistence

## Abstract

A possible solution is offered to help resolve the “two times problem” regarding the veridical and illusory nature of time. First it is recognized that the flow (passage) of time is part of a wider array of temporal experiences referred to as manifest time, all of which need to be reconciled. Then, an information gathering and utilizing system (IGUS) model is used as a basis for a view of manifest time. The model IGUS robot of Hartle that solves the “unique present” debate is enhanced with veridical and (corresponding) illusory components of not only the flow of time but also the larger entity of manifest time, providing a dualistic IGUS robot that represents all of the important temporal experiences. Based upon a variety of prior experiments, that view suggests that the veridical system is a reflection of accepted spacetime cosmologies and through natural selection begets the illusory system for functional purposes. Thus, there are not two opposing times, one outside and one inside the cranium. There is just one fundamental physical time which the brain developed, now possesses and is itself sufficient for adaption but then enhances. The illusory system is intended to provide a more satisfying experience of physical time, and better adaptive behavior. Future experiments to verify that view are provided. With a complete veridical system of temporal experiences there may be less need to reify certain temporal experiences so that the two times problem is less of a problem and more of a phenomenon.

## Introduction

### Two Times Problem

The ancient Greek philosophers began the debate as to whether or not time is an illusion. Currently, most physicists opt for Einstein’s view that the “past/present/future are illusions even if stubborn ones” (Davies, 2002). For some, time does not exist at a fundamental level but is derived (Barbour, 1994; Rovelli, 2011). Others present a spacetime cosmology that provides a mechanism to account for the “flow” that the brain undeniably experiences. Ellis (2014; also, Elitzur, 1992) proposes a universe that grows, the edge of which provides the passage (flow) that humans experience. Aerts’s (2018) theory of “Refounding Relativity” provides a method to account for the reality of “change.” Some philosophers such as McTaggart (1908) took an extraordinary position that logically time is an illusion. More recently, Price (2011) makes the case that the components of the flow of time (FOT), including motion and the moving present are subjective. However, philosophers of physics such as Broad (1923), and more lately Capek (1991) and Maudlin (2002) have made room in their metaphysics for objective flow. Fortunately, some philosophers recognize that “philosophical speculations need to be disciplined in the face of hard facts of neuroscience and experimental psychology” (Montemayor, 2013). Most recently, Callender (2017) examines the FOT carefully noting it is part of what he calls “manifest time” which includes the experiences related to the “now” and the past/future asymmetry. Then he suggests a parallel between the “two times” problem and Sir Arthur Eddington’s “two tables problem” which he views as two interpretations. One is a manifest table that looks and feels solid. The other is a scientific table composed of molecules in between which is much space.

Recently, Buonomano and Rovelli (2021) summarize the current disagreements between neuroscience and physics. The most concerning problem remaining for Rovelli is that of the “flow” of time which he attributes largely to entropy. By contrast, Buonomano questions the validity of the Block Universe. Both are understandably wanting to reify human time. Understandably, it is a terrible feeling believing that some of your perceptions are illusions. Perhaps the best and most succinct summation of the “two times problem” was provided when Gleick (2011) reported on the physicist Feynman’s view on the illusion of time.

“It seemed to Feynman that a robust conception of ‘now’ ought not to depend on murky notions of mentalism. The minds of humans are manifestations of physical law, too, he pointed out. Whatever hidden brain machinery created (one’s) coming into being must have to do with a correlation between events in two regions of space—the one inside the cranium and the other elsewhere ‘on the spacetime diagram.”’

On Richard Feynman, 1963

This is a concise phrase to contrast and relate human time to physical time. Feynman was asking for a physical explanation for the human experience of time. He felt that there must be a fundamental connection between the two times and that it would ultimately involve physics.

### Introduction of the Information Gathering and Utilizing System View to Solve the Problem

In response to Feynman the problem is approached here with a unified theory of manifest time (combined human temporal experiences). First, it involves explication of the IGUS “information gathering and utilizing system” view proposed by Hartle (2005, 2014). It mathematically demonstrates that the experiential past, present, and future are not properties of four-dimensional spacetime, but notions describing how individual IGUSs process information. For that reason, the conflict between physics and psychology for this particular aspect of temporal experiences should not, it says, exist. The potential problems with using a robot model or system as an experimental platform for the study of human behavior is reviewed by Datteri (2021).

Hartle’s IGUS view is widely accepted, amongst physicists at least, to bridge the gap between physics and psychology for issues relating to past/present/future and the “now.” Hartle provides a means to reconcile the physical “now” with the experiential “now.” He starts with the proposition that the world is four-dimensional according to fundamental physics, governed by basic laws that operate in a spacetime that has no unique division into space and time. He discusses the origin of this division (into present, past, and future) in terms of simple models of information gathering and utilizing systems ∼IGUSs. Past, present, and future are not properties of four-dimensional spacetime, but notions describing how individual IGUSs (robots) manipulate information. Their origin is to be found in how these robots were constructed. There is a localized notion of “present” at each point along an IGUS’ world line. But modes of organization that are different from present, past and future can be imagined that are consistent with the physical laws. Loosely speaking, it is being suggested that the past/present/future is outside of physics as is the case for music. With help from Shepard (1994) and Shepard (2004) he proposes a falsification test. He suggests it would be possible to construct IGUS robots that process information differently and therefore experience different “presents.” For example, a robot with a split visual system (SS robot) could experience the present with one half screen and events from the immediate past with the other half screen. So doing would confirm that there is no unique “present.”

**FIGURE 1:**
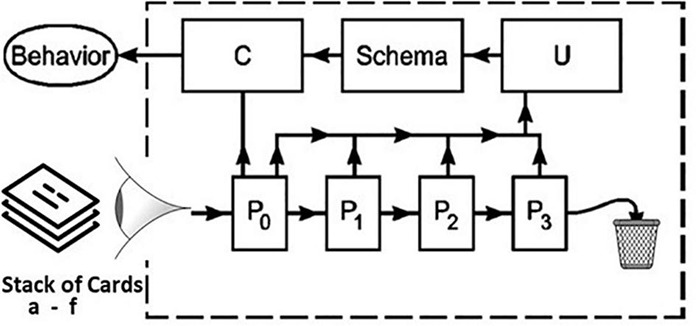
Schema for the model information gathering and utilization system (IGUS). At every proper time interval, the robot captures an image of its external environment. In this case, the robot experiences a stack of cards labeled *a*, *b*, *c, d, e, f*, etc. whose top member changes from time to time. The IGUS robot chooses how to route and utilize its information. Figure modified from Hartle (2005).

A simple schematic for the human (model) IGUS is given in Figure 1. At every proper time interval, the robot captures an image of its external environment. In this case, the robot experiences a stack of cards labeled *a*, *b*, *c, d, e, f*, etc. whose top member changes from time to time. The IGUS robot chooses how to route and utilize its information. The robot uses the images in registers, and in two processes of computation: C (conscious), and U (unconscious). The process U uses the data in all registers to update a simplified model or schema of the external environment. A schema of the external environment is used by C together with the most recently acquired data in to make predictions about its environment to the future of the data in, make decisions, and direct behavior.

In sum, the Hartle view allows one to appreciate the subjectivity of past/present/future that is said to be compatible with physics. Regarding the actual “flow” of time he attributes that to the movement of information in and out of the C (consciousness) register. In other words, the experiential flow component of the FOT is attributed to the utilizing system of the robot and not the time of physics.

### The Many Information Gathering and Utilizing System Views

That IGUS concept to deal with the “two times problem” was picked up by Ismael (2015, 2017). She augmented the IGUS by including “flow” and higher-level temporal beliefs—what are figuratively speaking “gadgets” to the robot. Hertzel (2016), Huggett (2018), and Dorato and Wittmann (2019) all share the view as to the importance of IGUS to explain passage (flow). Recently, Callender (2017, p. 227) provides a most needed expansion of the model IGUS and relies upon it for the “beginnings of a theory of time flow.” He augments the IGUS robot with even more “gadgets” to account for many other phenomena, including objective temporal experiences but also others such as “motion qualia” all of which come under the heading of “manifest time.”

Recently, we introduced yet another IGUS version but one involving a “dualistic model” (Gruber et al., 2020b). After acknowledging that the “present” component of the FOT requires an IGUS model for its explanation, it was postulated then that each of the other components of the FOT have both an illusory and non-illusory (“real”) aspect. That model is derived in part from 10 chosen spacetime cosmologies (see Table I of Gruber et al., 2020b). Many of the spacetime cosmological claims take Einstein’s Block Universe as a basis. Notable is the view that objects in the universe are really events that “happen” (Rovelli, 2011, 2018). In other words, the Block is not “frozen” as originally interpreted. Some spacetime views modify the Block to allow it to grow and thereby provide an objective basis for temporal “flow” (Ellis, 2014). All the experiences of the FOT are then contrasted to what these spacetime cosmologies have to say about those very same phenomena. Then it is possible to construct a dualistic mind model based upon Hartle’s IGUS robot. Without a doubt there will be controversy here simply because the 10 spacetime views are not unanimous and it is necessary to decide which ones will most likely be correct and sustained.

## A Dualistic Information Gathering and Utilizing System View of Manifest Time

The dualistic model needs expansion to all major components of manifest time. A theory is said to explain a wide array of phenomena and then make predictions. Therefore, we can start with established principles from both disciplines of physics and cognitive science. From them extract or introduce a more comprehensive view (a theory some might say) to explain and subsequently predict. The following two are chosen.

### Two Principles for a Dualistic Mind Approach

1) *As an information gathering and utilizing system (IGUS), the human has an experience of past/present/future that is consistent with the physical laws.*
2) *The phenomenon of dynamism is an experimentally demonstrable illusory experience.*

Principle #1 is supported by Hartle (2005) and principle #2 by Gruber and Block (2017), Gruber et al. (2018). These two apparently contradictory principles suggest that one principle cannot be denigrated in order to preserve the other. A way to reconcile them is to consider the possibility that one exists to supplement or augment the other. By analyzing known veridical and illusory components of passage a dualistic classification is derived and in turn more veridical components of flow (passage) are found as will be described below. Furthermore, it is argued that the veridical system begets a corresponding illusory system of temporal experiences. That dualistic construction is then applied, in the form of add-on “gadgets” to Hartle’s “model IGUS” (representing the human) resulting in a dualistic IGUS that represents manifest time. It is argued that the illusory system’s sole purpose is to enhance the human experience of time. It is said to be the product of natural selection. The net result for the two times problem is that there may less of a need to reify the “flow” of the FOT. Knowing that there is a complete veridical system might lessen the need to deny aspects of spacetime theories that stand on solid ground. Finally, knowing that the illusory system is, if necessary, dispensable should soften the perception that our brains have been left out of the physics of time.

### Definitions

Before explication, it is necessary to avoid confusion by defining terms which have often been conflated during the past debates involving the “two times problem.” The term “dualistic” means nothing more than that there are two types of experiences (or cognitions) for each component of manifest time including the flow of time (FOT). The term is used similarly by Vallerand (2015) to denote two types of passion: a harmonious and an obsessive passion, the former being healthier. The term “dualistic” should also not be confused with dualism—a view in the philosophy of mind that mental phenomena are, in some respects, non-physical, or that the mind and body are distinct and separable. Dualistic is also not to be confused with the important discovery of phylogenetically and dual temporal cognitive systems (Hoerl and McCormack, 2019). It is also not the dual model in philosophy of time that distinguishes conscious from unconscious time perception based on agency which is used to clarify the metric from the subjective requirements of time cognition (Montemayor, 2017a,^2019^).

The term illusion refers to a perception that has no basis in reality^1^ which in turn, defers the problem to what the currently accepted laws of physics suggest. It has long been an ambiguous term which needs defining (Buonomano, 2017). Consider the perceptual completion that the brain provides to fill in the retinal blind spot. On the one hand, “filling in” is illusory. On the other hand, the brain guesses correctly. One would say that the “perceptual completion” of that perception is not an illusion; it provides no false information the way a mirage does. It provides helpful information and is more accurately referred to as a “perceptual add-on,” one that is veridical (not contradicted by accepted physics). When only cognition is involved such as a myth or belief it can be referred to as a cognitive add-on. However, the need to make a distinction between the terms cognitive and perceptual is not critical as Mroczko-Wasowicz (2016) questions the close relationship between the two. The word illusion is retained here because it is engrained in the literature and less cumbersome than “perceptual add-on.” Lastly, there is the new term, “manifest time” of Callender (2017). It is the sum of human temporal experiences that are readily perceived and recognized by the mind. It includes experiences associated with “subjective time” and the flow (passage) of time. It is meant to be a complete collection of what is perceived. All major dualistic components of manifest time will be elaborated upon here. The three commonly associated with flow (passage) are: (1) a unique (moving) present, (2) dynamism of change/motion, and (3) directionality (temporality).

## Dualistic Components of Manifest Time

### ”Present” [Unique (Moving) and No Unique Present]

**FIGURE 2:**
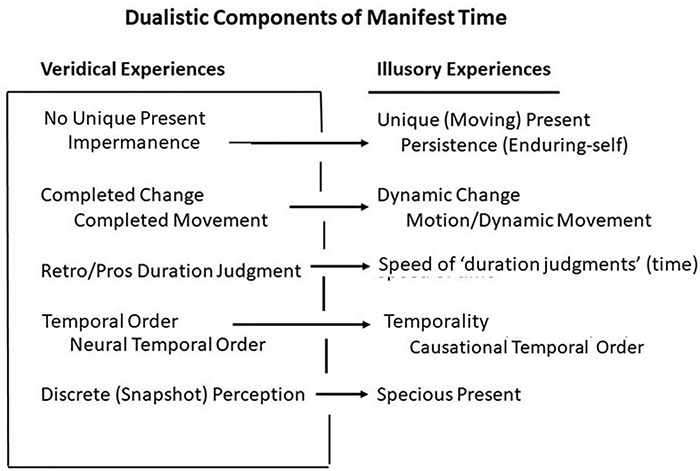
Major components of manifest time. Veridical components beget illusory components.

Of the common components of flow, one of the most hotly debated ones is the alleged unique “present.” It is first from a list of several components of manifest time (Figure 2). To experimentally confirm that the present is not unique Gruber and Smith (2019) chose to test Hartle’s IGUS hypothesis that a new “present” can be fabricated suggesting that the current one we humans possess is not unique. The experiment involved the construction of a split screen (*SS)* robot using a VR headset screen containing two “presents” of slightly different local time intervals. However, upon construction, it was immediately apparent that the observer did not experience two simultaneous “presents” because there was no sense of immersion in the environment. To create this immersion experience, a similar robot was created in which the observer is permitted to alternate between “past” and “present” screens *ad libitum*—the Intermittently Behind (IB) robot. By being able to switch between equally realistic time periods, the observer experienced what was intended in the split screen (*SS)* robot except in an alternating instead of a simultaneous manner.

To ascertain the experience of “presence” in past events the participant was asked whether she agreed or disagreed with specific statements: (1) “seeing the event again was just as real as the first time” and (2) “during VR replay of the second event it seemed like I was ‘there.”’ Participants unanimously agreed with both statements. This type of post-experiment questioning is more revealing than simply asking if there was a feeling of being in the “past” because no participant has ever been in the past. The participant was also allowed to go back and forth between “past” and “present” *ad libitum* by pressing a button. Uunsolicited participant comments that it clearly felt like “being in the past” were received. A few participants even indicated that they sometimes “got lost” between what was “past” and what was “present.”

**FIGURE 3:**
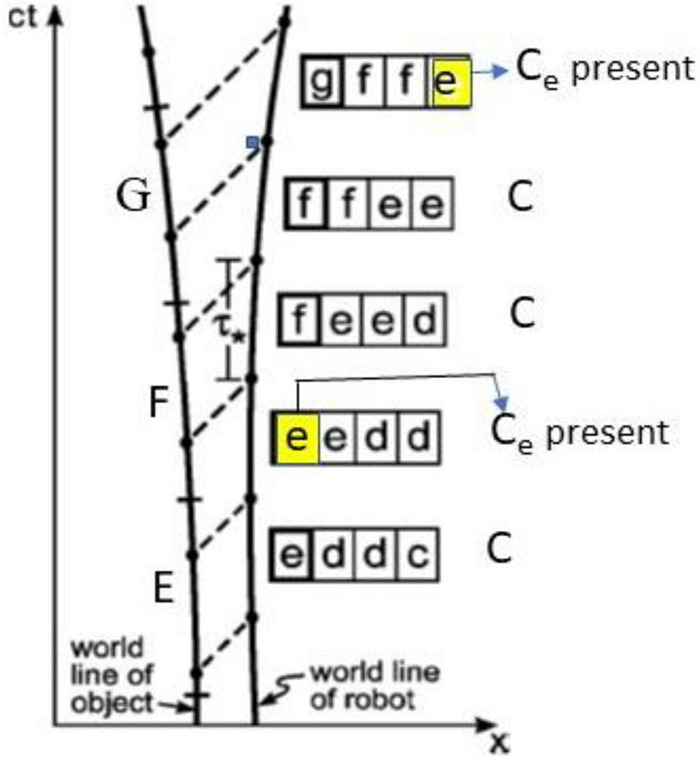
A world-line description of the *intermittently behind (IB)* robot.

A worldline description of the “present” for an *IB* robot is given in Figure 3. It shows the worldline of an external object that is the source of its images such as the stack of cards in the prior figure. This source changes its shape at discrete instants of time delineated by ticks, passing through configurations c, d, e, f, and g. As an example, the object E is recorded as e in two adjacent registers—thus e, e. The number of registers for e is simply proportional to the duration of observation. The image in each register is then experienced as C_e._ These e’s are experienced again as C_e_ when each e moves to another register that is further away (along the world line). In short, the robot is permitted to utilize information as it chooses. In this case the present from register *e* is experienced at two different points along the worldline, i.e., the IB robot experiences the *same present twice*.

It should be noted that some of Hartle’s IGUS robots do exist in humans to a limited extent (Gruber, 2008). There is an Always Behind (AB) Robot. This robot has input to C computation only from a particular register P_K_ > 0, and the schema. That input is thus always a proper time behind the most recently acquired data. The (AB) robot also would have a tripartite division of recorded information. Its present experience would be the contents of the register P_K_. It would remember the past stored in registers P_K+1_, …,P_n_. But also, it would remember its future stored P_0_,…P_K–1_, at a time ahead of its present experience. In other words, its answers to questions about “what’s happening now?” would be slightly out of date. However, it would have premonitions of the future. Another IGUS example is a savant, the No Schema (NS) robot that has input to *C* computation from all the registers *P*_0_ – *P*_*n*_ equally. It employs no unconscious computation and constructs no schema, but rather makes decisions by conscious computation from all the data it has. Savants process almost every piece of incoming information and recall it as immediately and as vividly as information in the present. However, an *NS* robot would make inefficient use of computing resources by giving equal focus to present data and data from the past whose details may not affect relevant future prediction.

Verifying Hartle’s prediction suggests that the brain has as one of its fundamental experiences of manifest time an experience of a potentially variable past/present/future and an illusory unique present. To be clear, although it was possible to construct a robot other than the “model robot,” the IB robot does not prove that the “moving present” is an illusion. It only establishes that there is a notion of a “present” at each point along the worldline. The actual “moving present” is a dynamistic illusory experience that is more related if not identical to the experience of “moving”—in other words “motion” which is described as an illusion below. In agreement, Romero (2015) indicates that for physics “there is no ‘moving present’ only an ordered system of events.”

### Persistence (Enduring Self) and Impermanence (Ephemeral Self)

For years the flow of time (FOT) debate revolved largely around two FOT levels: the past/present/future illusion, particularly the “moving present” (the upper level) and the dynamic temporal experiences such as motion (the lower level) (Gruber et al., 2015). However, another related phenomenon is made more evident by Ismael (2011) and Price (2011, p. 35) who discovered what they would refer to as a *double illusion.* It turns out that the subjective or illusory phenomenon of the FOT rests upon another illusion that in some sense, they say, is more important. It is the phenomenon of *persistence*, specifically, the “persisting self” (Ismael, 2007; Ismael, 2011). Under the name of “enduring self” Paul (2016) reviews this matter thoroughly. A human needs to feel that she persists and is not simply a conglomerate of impermanent events as spacetime cosmologies suggest. The observer, in a unique (moving) present, wants to believe she is a single individual and not multiple momentary individuals extending backward in time.

In the Block Universe, persistence of that sort has no place. When consoling the wife of his best friend Besso who had just died, Einstein said, in effect, that Besso was still there (Elitzur, 1992; Davies, 2002). The implication was clear: there is a Besso who may be dead but another who is alive is in the past. Clearly, this time illusion is difficult to accept for most. However, it is much easier to acknowledge impermanence as veridical if one happens to hold the spacetime view that the universe is composed of events, and that the observer, too, is basically a series of complex events (Romero, 2015; Rovelli, 2018). This issue of persistence as an illusory phenomenon in the physics of Minkowski spacetime is reviewed by Balashov (2007). The two opposite views regarding persistence are known as endurantism (or three-dimensionalism) and “perdurantism” (four-dimensionalism). According to the former, objects are extended in three spatial dimensions and persist through time by being wholly present at any moment at which they exist. On the latter, opposing account, objects are extended both in space and time, and persist by having “temporal parts,” no part being present at more than one time. Only perdurantism is compatible with spacetime physics and suggests that object persistence is not veridical.

With a hypothesis that experiential persistence is an illusory experience, a pilot experiment was performed to demonstrate that it can be precluded (Gruber et al., 2020b). Observers (“human IB robots”) wearing the backward-in-time VR apparatus were allowed to watch a remote controlled toy dog roaming about as they went “back and forth in time.” They lost the experience of persistence. Going back and forth into the past (e.g., 30 s back) she would note a moving toy dog. When she was in the past, she might see the dog to her right even though it was actually located to her left. When she returned suddenly (in a fraction of a second) to the present she would see the dog to her left where it actually is. The experience was that the dog did not appear to be the same dog because it could not have traveled several feet that quickly. The explanation is based upon the principle of spatiotemporal priority which occurs for the well studied phenomenon of “object persistence” (Scholl, 2007). When deciding whether an object is the same persisting object from some earlier time, factors relating to how and where that object has moved will almost always trump factors relating to what the object looks like. For example, when a car goes through a tunnel it is considered to be the “same” car if there are no major changes to it and if the time of exit is appropriate for the entering velocity. If it exits almost the same time as it entered it is “not same.”

Not all share the view that persistence is illusory, and that impermanence (ephemerality) is veridical (reviewed by Haslanger and Kurtz, 2006). However, it would seem that any argument on behalf of persistence likely stems from a desire not to be ephemeral, i.e., not to be a fleeting individual. In fact, it is more likely that the whole adaptive purpose of persistence illusion is to provide a singular “self” that can move along with the “present.” Other philosophers do support the physics view. Paul (2010) takes the position that persistence, just like apparent motion. is an illusion, and refers to it as “apparent persistence.” The illusory cognitive add-on of a persisting or enduring “self” allows the observer to either view herself as stable with events and the “present” moving by relative to her, or vice versa, that she is moving relative to events of the environment. Callender (2017) considers the “self” important enough to add it as a gadget to the IGUS. The entire phenomenon of self and its illusory aspects are beyond the scope of this paper but are reviewed by Klein (2004), Blackmore (2012), Hood (2012), and Velleman (2016).

It is worth noting that a case can easily be made that the veridical impermanence is a viable alternative to the enduring self. There is a large group of people who opt for the view that the self is not persistent or at least should choose not to be persistent. Outside of Western civilization there are those who subscribe to that view. It is a belief amongst those adhering to Buddhism (Struhl, 2020). It is a belief that the individual is really, or at least should consider herself to be “ephemeral” (impermanent). Recognition of the dualistic nature of this particular temporal phenomenon may help legitimize the self-illusion (enduring self) and foster a reconciliation with the Eastern view of self.

### Change (Dynamic and Completed)

It was not that many years ago that “change” was discovered to have dualistic experiences (Rensink, 2002). It was definitely not realized until recently how important that understanding of the change phenomenon would be for the two times problem (Gruber et al., 2020b). There is “dynamic change”—the experience of seeing an illusory change occur such as one color or shape to another. There is also “completed change”—the non-illusory experience that the change “must have occurred.” In a flicker paradigm Hollingworth (2008) demonstrated it with an initial object (such as a cup) and a different object (another cup) for 250 ms duration each and a blank interstimulus interval (ISI) varying from 200 to 5,000 ms. Participants reported a strong impression of “seeing the change occur” at 200 ms, a weaker impression at 1,000 ms, and no impression (completed change) at 5,000 ms. After hypothesizing that the experience of “happening” was part of the “flow” in the FOT phenomenon Gruber and Block (2013; 2017; see also Gruber et al., 2020a) studied the change of more prolonged, featural processes such as bread toasting and noted the ISIs at which dynamic and completed change occurred. In several sensory modalities (auditory and tactile) the phenomenon was expressed by the participants as experiencing the scene (events) to actually “happen” (dynamic change) vs. knowing it or “not seeing it actually happen” (completed change).

Dynamic change is one of many elements of dynamic perceptual completion (DPC) all of which provide the all important experience and phenomenon of temporal continuity for discrete or interrupted perception. Dynamic change and all DPCs are perceptual add-ons. They do not necessarily provide significant information for the observer other than to indicate (loosely speaking) that there are multiple events of unspecified type in between two temporally adjacent stimuli. Were there no experiences of DPC, however, it is likely that the brain would cognitively deduce it (i.e., continuity) anyway.

Physicists have tried for years to reconcile the phenomenon of “change” in the “frozen” Block Universe. Both Rovelli (2011, 2018, p. 97) and Aerts (2014, 2018) have successfully “unfrozen” it. The “change” in their cosmological theories is said to be real, and most physicists today have adopted that view. When the dualistic view was first introduced (Gruber et al., 2020b) the hypothesis then was that a dualistic temporal experience of “change” should be expected, in particular a veridical one for physics. The claim then and now is that completed change represents the “change” in physics. A dynamic change simply augments that experience. The fact that the human brain also evokes an illusory form of change does not conflict with its “real” aspect.

When analyzing change, it eventually becomes necessary to delve into the phenomenon of “becoming” because of the intimate relationship of the two. Philosophers and physicists have debated the specifics of becoming and its relation to change for some years now (Mellor, 1998). Also, Savitt (2002, p. 567) argues that Special Relativity has within it two concepts of time: (1) coordinate time and (2) proper time, “the latter being a kind of time perfectly apt for ‘becoming.”’ From a physics viewpoint, Aerts (2018) points out that the apparent contradiction between a process view on reality (where there is a being and a becoming), and a geometrical view (where there is only a being and no becoming) need not exist; therefore, change is said not to be an illusion. In sum, the phenomenon of becoming can and should be recognized; but for purposes here it need not be treated as a separate component of manifest time because the phenomenon of change is dealt with in depth.

### Motion (Dynamic Movement) and Completed Movement

When the dualistic model was introduced one of the hypotheses included the notion that it should be possible to discover a corresponding veridical experience for every illusory one. That would not only include change, as just demonstrated, but it would include its most important aspect—motion. Motion is divided into “real” and apparent types. Briefly, apparent motion involves both beta (movement of an apparent object between successive stimuli) and phi (objectless movement) (Steinman et al., 2000). However, the experience of “real motion,” itself, is considered to be an illusory percept for the following reason. Visual perception is generally agreed to be discrete at a rate of 10–13 Hz, with the continuous wagon wheel illusion. As the speed of the wheel increases, a point is reached when it starts to reverse itself—an illusion due to the phenomenon of aliasing (VanRullen and Koch, 2003; VanRullen et al., 2010). It must be acknowledged that a couple of studies are skeptical that discrete perception has been proven by the wagon wheel experiments (Kline and Eagleman, 2008; Holcombe, 2014). Assuming that perception is discrete, Koch (2004, p. 274) suggests that motion is “painted on” to each frame. At this time, it is uncertain if that “paint” is phi, beta or both.

Fortunately, the world is quite navigable at a perceptual rate of 13 Hz (although not always comfortably) without the DPC of motion. For example, a bird in flight flashed on and off to the hunter’s eyes at 10–13 Hz has a staccato type of appearance. However, the bird is visible enough to the hunter such that an accurate shot is possible. The superimposed motion experience can be thought of as a perceptual add-on, one that augments the perception of movement. Specifically, beta may be filling the gaps with images of the bird and in that sense is veridical because the brain guessed correctly. On the other hand, it implies continuity and persistence which is not veridical. There is also one caveat, in that motion would seem to be essential when considering the debilitating life of akinetopsia patients. However, these patients are missing more than that type of motion (such as beta or phi). Their perception is erratic with many frozen, prolonged intervals (Rizzo et al., 1995). By contrast, the patient with cinematographic vision (a flickering series of stills) from a seizure disorder or migraine (Sacks, 1999) may get all the essential information regarding spatiotemporal changes.

Similar to the situation described above with change, motion (also termed *dynamic movement*) is the illusory counterpart of a (veridical) *completed movement*. An experimental example of it in the form of positional change was given by Nakashima and Yokosawa (2012, p. 269) in a flicker change detection task. A 250 ms duration image of a bed oriented to the right was alternated with another oriented to the left with black (blank) ISIs between them. As the ISI increased, that impression (of bed rotation) weakened and was lost after 1,000 ms. Then participants experienced what can be considered to be *completed movement*, i.e., “not seeing the change occur.” The similarity to completed change is noteworthy.

From the viewpoint of physics motion is denied in the Block Universe, “End of Time” and the “Order of Time” spacetime cosmological views. Physical continuity is not in the cosmological scheme. Instead, what is expected by them is that events, including cerebral events, be discrete. It is no coincidence, therefore, that the temporal experience of “completed movement” is a discrete process as is completed change. These two veridical experiences are good examples as to how much physical time does reside within the human cranium. Meanwhile, the illusory, dynamistic aspect of movement (i.e., motion) satisfies the desire of spacetime theorists such as Dowker (2014) with her Spacetime Atoms view to “breathe life” into the Block Universe. However, it is coming from the illusory system within the cranium, not necessarily the edge of an expanding universe. Therefore, we add it as one more “gadget” to the dualistic IGUS.

### Temporality and Temporal Order

The initial dualistic model also hypothesized that temporality should have a veridical and illusory aspect (Gruber et al., 2020b). Of note, temporality is defined in many ways. Physics and the psychological sciences often define it as the totality of time experiences within their discipline. Here the term is restricted to the before/after human experience. Ruhnau (1997) and Pöppel and Bao (2014) provide the basic analysis of that experience in the first several seconds. Experienced time is segmented into a hierarchy of domains or zones. For intervals of 5–30 ms there is no experience of before/after. It is the “atemporal” zone. When put together those ultra-brief zones provide a longer 2–3 s zone that compromises the human experience of “present (nowness).” Each 2–3 sec of “nowness” is then linked with the previous one, but the continuity of that entire experience is said to be an illusion (Pöppel, 1997; Ruhnau, 1997). Further analysis of that hierarchy of temporality is provided by Montemayor and Wittmann (2014) who emphasize that the continuity of experience (requiring working memory) involves multiple seconds to generate a platform for the narrative self. It is important to note that these intervals comprising the temporality experience are part of the “succession of experiences” (Arstila, 2016). Moreover, there is an additional overlaying “experience of succession” [also known as the “feeling of succession” (Hoerl, 2013) for at least the first three zones].

The illusory component of the temporality experience is brought to the fore by Arstila (2018) who insists that “*the succession of experiences and the experience of succession are two different things*.” Historically, James (1890, p. 628–629; see also Block, 1994; Block and Patterson, 1994) expressed this view similarly as: “A succession of feelings, in and of itself, is not a feeling of succession. And since, to our successive feelings, a feeling of their own succession is added, that must be treated as an additional fact requiring its own special elucidation.” Plainly put, temporality comprises both the experience of succession and the succession of experiences.

To argue that there is an illusory aspect to temporality consider the thought experiment of C.D. Broad (1923; see also Montemayor, 2009, 2012; Arstila, 2016). When looking at the big hand of a clock there is an experience of succession and also a succession of experiences as it moves along passing the numbers on the dial. By contrast, when viewing the little (hour) hand there is no experience of succession because the movement is imperceptible. However, there is still an experience of a succession of experiences when it eventually passes a number. Thus, one is left with temporal order.

It is helpful to contrast the experience of succession to that of motion. Arstila (2016, 2018) notes that both are experiences that are said not to extend time and are part of his “snapshot theory.” He refers to the illusory experience associated with “pure motion” and then notes that the same can be said of succession, referring to it as “pure succession.” Both serve the same purpose for human adaption by providing a continuity for events and an assumption of “sameness” or persistence which is contrary to the physics view.

There is another temporal order phenomenon that is dualistic. The brain is constantly rearranging the timing of events for consciousness. Recall, for example, that the neural arrival times for the auditory and visual components of snapping fingers differ. The temporal order differential needs to be adjusted closer to but not necessarily zero if they are to be experienced simultaneously. But, that is a minor, easily correctable problem for the brain. A more serious situation is the temporal order differential between a decision to act and the associated motor response (such as a key press). The decision to key press must precede the press itself (Eagleman, 2008). Proper cause and effect are required. In essence, there is an illusion that we live in the immediate present. Whereas events are experienced relatively quickly after arriving at the sensory cortex and experienced soon afterward, some processing particularly that of awareness may not occur for approximately 500 ms later. That is an astonishingly long period of time. Ordinarily this would cause an individual to live in the past, and not feel she has free will. It would appear that awareness of motor function is (postdictively) moved backward in chronological time so that the individual can experience cause and effect and feel she is in charge, i.e., exhibits free will (Libet, 2004). The topic of temporal order judgment and free will is reviewed by Shimojo (2014).

The postdiction process modifies the veridical “neural temporal order” to provide what can be called an illusory “causational temporal order” that enables the observer to believe she is in charge. Living without the benefits of postdiction is not common but is doable. The brain can simply accept the fact that the body is thankfully acting on behalf of the conscious individual. For example, professional runners are known to experience their legs take off from the starting blocks before hearing the gun. It is of no concern to them. It’s not much different than stepping on a tack with a reflex of retracting the foot and a feeling that one need not be in control of everything. The existence of free will is a hotly debated beyond the scope of this paper. The compromise view of it is provided by Haggard (2019). The point here is that a gadget for “neural/causational” temporal order is another addition for the dualistic IGUS robot.

### Speed of Time and Duration Judgments

Totally unrelated to temporal order but also important is the veridical phenomenon of duration judgment and its corresponding illusory experience—the “speed of time,” which can be thought of as the speed of duration judgments. The phenomenon of duration judgment has been studied thoroughly with its prospective and retrospective types (Block and Zakay, 1997; Wittmann, 2016; Montemayor, 2017b). Prospective duration judgment involves some sort of timing mechanism. The original “internal clock” has not been discovered (reviewed by Block, 2003) but “population clocks” are a likely answer (Buonomano, 2017). These judgments are also influenced strongly by attention and cognitive load (Block et al., 2010). Retrospective duration judgment involves memory, and in particular the memory and contextual cues of those events (Block et al., 2018), all of which provide objective duration measurements even if not necessarily accurate.

The corresponding illusory experience, the speed of time, is judged when asking “how fast time went.” And, it can be thought of as how quickly the events went by. Droit-Volet and Wearden (2016) studied this phenomenon referring to it as the “passage of time judgments.” This temporal experience was assessed by asking the participants to indicate how quickly time seemed to pass during a task, an experience that is different than its duration. Their results showed that although an interval can be retrospectively underestimated (such as sitting in a waiting room), time can be judged as passing slowly during that interval.

### Specious Present and Discrete (Snapshot) Perception

Unlike the obviously needed temporal parameter of duration there is a very well known phenomenon of time that has not until recently been viewed as a temporal experience for the “two times” debate. The specious present is the interval of time in which one’s perceptions are considered to be in the present. All the notes of a bar of a song seem to the listener to be contained in the present. For example, the four most famous notes of classical music are from Beethoven’s Fifth, and not coincidentally are played with an approximately 3 s window providing a single, complex unified experience above and beyond the individual notes. Extending notes beyond that duration causes the emotional impact to be weakened because the bubble of the experience would be broken. If nothing else, that special 3 s interval involves immediate memories that are relatively intense making it easier for example for a quarterback to know where his receivers were located a second or two ago before deciding where to throw the football.

The duration of the specious present varies widely. James (1890) suggested that the duration may easily be 7 s, whereas Dainton (2000) believes it is closer to a half second if one measures it by the duration of experiential succession between events. Pöppel and Bao (2014) studied it in great detail noting that there are a number of cerebral functions that can only be temporally integrated during that interval. It is then that temporal continuity is applied by the brain to the events, allowing it to successively negotiate those events and integrating them. “Without this continuity, the brain would be lost in a jungle of unrelated pieces of information” (Pöppel, 1997, p. 117). He proposes that temporal integration up to 3 s is a general principle of the neuro-cognitive machinery. The temporal limit is said not to be determined by what is processed, but by intrinsic time constants.

Philosophers weighed in notably Grush (2005) with a compelling phenomenological view of temporally extended time (the trajectory estimation model) to explain how a group of memories is perceived in the present moment. However, Arstila (2018) challenges the assumption that the contents of our experiences embrace temporally extended intervals of time. He argues that the specious present doctrine is false and presents a theory that does not require the notion of a specious present. Lastly, it is quite likely that some animals do not have a specious present although it is difficult to imagine how that would be tested. With respect to the view from physics, the notion of an extended present is, understandably not congruent with the “present” of Hartle’s robots and Minkowski spacetime.

### Flow Whoosh and Dynamism

Until now little has been said about (1) “flow” in the “flow of time,” (2) the dynamic aspect of temporal experiences often referred to as “whoosh,” and elsewhere (3) the “feel” for Paul (2010). The exact mechanism behind this dynamic experience is debatable. Callender (2017, p. 255) submits that “… to make our IGUS believe that time *whooshes* by we need it to represent itself in its model of the world as an enduring self.” By contrast, flow is the dynamic experience of time that seems to mostly concern Rovelli (Buonomano and Rovelli, 2021) who attributes it to entropy. It is his principal issue for bridging the gap between physics and neuroscience. In so doing he is attempting to reify that particular human experience. It is conjectured here that “flow” represents the dynamism of a few temporal experiences from the illusory system, e.g., motion (dynamic movement), dynamic change, and the “feeling of succession” (“pure succession”) of temporality. In the final analysis these experiences are ones of continuity and implied “sameness” all of which find no home in most spacetime cosmologies.

### Summary of the Dualistic Mind Theory

Now that many “gadgets” have been added to the model IGUS, the dualistic robot with all of the significant parameters of temporal experiences is largely complete. There are a few others Callender (2017), but space does not permit reviewing them. Also, until now nothing is mentioned as to how these temporal processes might be implemented. A general discussion of such temporal process is reviewed by Marchetti (2014) but is beyond the scope of this paper. However, in keeping with the goal of helping to solve the two times problem, the IGUS model for the flow of time is expanded to a dualistic view of manifest time. The “gadgets” figuratively represent the many components of the flow of time and other temporal properties of manifest time. Moreover, the components fall into a veridical system that is compatible with modern spacetime cosmology and a corresponding illusory system that augments the veridical experiences. The dualistic mind approach avoids implicating many human experiences of time as illusory and also removes the compulsion to insist that the experiential “flow” is real or veridical. Both systems of veridical and illusory experiences exist, and are not only not in conflict, but they guide adaptive behavior. The two times problem is thereby offered as a possible solution.

To be transparent there are certain weakness with the dualistic mind view. For one, it ignores the auditory and somatosensory perception by the dualistic IGUS. The dualistic view does not explore, in depth, the phenomenon of precognition/premonition or déjà vu, temporal experiences that are almost universal other than the one method mentioned in the Always Behind (AB) robot. Also, by almost exclusively covering results from visual perception but not motor movement, somatosensory and auditory perception, etc., the model is admittedly biased toward vision. If one wanted to refer to Hartle’s IGUS robot as a visual robot no good argument could be given other than the fact that Special Relativity and Minkowski’s flat spacetime, of necessity, require photons of light for the IGUS robot to communicate. Lastly, it should be pointed out that the IGUS robot of Hartle that is expanded upon by others and now us should not be considered a working mental model. That would be inappropriate.

## Dualistic Information Gathering and Utilizing System Claims and Predictions

### Evolution of Information Gathering and Utilizing Systems

Resolving the unique/no unique “present” issue itself is not enough for Hartle (2005). He also suggests that the small variety of IGUS robots are involved in evolution (in a very general sense) with the most successful IGUS being the “model IGUS” representing most humans.^2^ A less successful robot is the NS (“no schema”) robot. As noted above it is a great conversationalist but wastes too much energy. Another is the AB (“always behind”) robot that is slow to respond to its environment but an evolutionary possibility. Although the model IGUS is best it has a meager structure of temporal properties. If one is to construct an actual evolutionary story for the model IGUS robot it would have to have had objective sensors at the outset in order to engage with the external world, acquire the necessary information for utilization so that it could direct all behavior. Those sensors of objective parameters would include timing, movement, and a detector for temporal order,—all the parameters that the acceptable spacetime cosmologies expect. For example, a means of detecting duration judgment would be necessary, something that is functionally similar to the hypothetical internal clock. Also, movement detectors, functionally similar to Reichardt detectors for visual motion (movement), would be necessary even if more primitive as is the case for animals in a lower part of the phylogenetic tree. In short, the IGUS would be expected to acquire the ability to evoke all of the major temporal experiences in the veridical system described above and a few more that Callender (2017, p. 261) suggests.

The benefits of adding components (“gadgets”) to provide illusory aspects of manifest time is substantial. For example, whereas Rovelli’s (2018) “Order of Time” expects a monitor for order (physical order), the early model IGUS would enhance that by begetting the illusory aspect of temporality (“pure succession”) as a part of the evolutionary changes to provide continuity. In the case of movement, the gadget of motion also provides continuity, and with it the experience of “sameness” or object persistence. More important, the evolution of persistence (the enduring self), the deepest illusion, provides an indispensable foundation for human personality. Without an enduring-self one cannot engage in mental time travel. It is the capacity to mentally reconstruct personal events from the past (using episodic memory) as well as to imagine possible scenarios in the future (episodic foresight/episodic future thinking). For reviews see Suddendorf and Corballis (2007), Klein and Nichols (2012), and Corballis (2013).

Most animals lack that ability and are not able to foresee and plan the future. Most, but not all, do not exhibit self-awareness of an enduring self. Although a crude test, many fail the mirror test in which a spot is placed on the front of their head, and they are allowed to look at a mirror to see if they exhibit self-awareness behavior (Hongshang, 2005). These animals assume that the one in the mirror is conspecific (of the same species but not the “same.”) causing them sometimes to react violently. Admittedly, some of these self recognition tests may be difficult for humans to interpret in taxonomically divergent animals, especially those that lack the dexterity (or limbs) required to touch a mark. For example, it has been possible to demonstrate that a fish, the Labroides Dimidiatus, shows behavior that may reasonably be interpreted as passing through all phases of self-recognition such as social reactions toward the mirror and frequent observations of their reflection (Kohda et al., 2019). In short, some self-awareness should be expected from a variety of animals.

### Consequences of a Loss or Dysfunction of the Illusory System

In addition to the positive contributions by an illusory system there is a potential negative effect due to its absence or dysfunction. This should be expected because it is seen following the loss of other illusory experiences that the human brain evokes for non-temporal experiences. For example, the patient with amusia from a stroke, particularly if she is a musician can be devastating (Sacks, 2008) in contrast to the individual who has congenital amusia and never experienced the aesthetics of music to begin with. Consider now the possible harm from a reversion from the “enduring self” to the “ephemeral self” (the impermanence of physics) as occurs in schizophrenia. In general, dysfunction of manifest time is a particular problem for some of these patients (Mishara et al., 2014). Those disturbances of the self are related to alterations in time processing which includes temporal order and temporal continuity (Giersch et al., 2013; Giersch et al., 2016). The “self” is normally experienced as being continuous in time. If not, it becomes a “self-disorder.” Martin et al. (2013) proposed that disorganization in time might impact patients’ ability to experience themselves as a continuous self, i.e., the patient may not feel that she is the same person at all times. For more see Montemayor and Wittmann (2014). The following excerpts are from an interview of a schizophrenic patient with a self-disorder (Fuchs, 2013, p. 84).

“Time is also running strangely. It falls apart and no longer progresses. There arise only innumerable separate now, now, now— quite crazy and without rules or order. It is the same with myself. From moment to moment, various ‘selves’ arise and disappear entirely at random. There is no connection between my present ego and the one before.”

A few humans with brain damage (particularly to the hippocampus) have been described as no longer being able to time travel. As a result of not traveling into the future the person is devoid of aspirations and wishes (Klein and Lofthus, 2002; Andelman et al., 2009). In one of their patient’s words: “I take each day one at a time. I don’t see past today and tomorrow. and can’t picture myself in anything beyond the immediate present.” Patients with that type of hippocampal damage cannot imagine anything they are likely to do on a subsequent occasion. They seem to be living in a “permanent present” (Tulving, 1985).

### Testing the Dualistic Mind View

A theory should not only explain a major phenomenon but make predictions. Just providing a mental model and expecting it to work would be insufficient. The dualistic view begins by invoking a number of hypotheses which were subsequently tested (Gruber et al., 2020a). Most important is the hypothesis that a complete veridical system contains all of the important temporal experiences to sustain the human for adaption. That includes veridical change as turned out to be the case with “completed change” and also veridical movement which was discovered in the form of “completed movement.” The Hartle hypothesis that a past/present/future notion is, in and of itself, consistent with Minkowski spacetime physics was verified.

Regarding predictions, more evidence is expected indicating that there is a complete physical system for temporal experiences independent of the illusory system. For example, although it is clear that completed movement exists it would be helpful to demonstrate that under some as yet to be described circumstances a human can function with all visual perception exhibiting only completed movement without dynamic movement (motion).

The dualistic view predicts an existence of a discrete (snapshot) perception in the absence of the specious present. A possible experiment to consider is as follows. When a series of visual images, e.g., a walking scene, with a large interstimulus stimulus interval (ISI), e.g., much greater than 3 s, is presented a snapshot-like experience is expected, devoid of not only motion and “happening (which has been demonstrated — Gruber and Block, 2017) but also the specious present.

Perhaps the most important prediction is that temporal order in the absence of the experience of succession (“pure succession”) can be sustained and is sufficient for adaptation. Currently, the thinking from the clock thought experiment is that somewhere between seconds and an hour of constant observation “pure succession” drops out and temporal order remains. An experiment similar to others that tests for the upper and lower limits of temporal order could be done. By varying the interstimulus interval and/or other parameters a point is expected to be reached when only temporal order of temporality exists. Lastly, one other prediction relates to the pilot experiment involving VR to demonstrate that the experience of persistence is illusory. It needs full experimental verification.

## Conclusion

An attempt to help solve the two times problem begins with the introduction of the original IGUS model that partially resolves the two times problem. It demonstrates that the experiential past, present, and future are not properties of four-dimensional spacetime, but notions describing how individual IGUSs, including humans, process information. The IGUS model is upgraded by others and now by our extended version to a dualistic IGUS or dualistic mind. It is suggested that “gadgets” representing components of manifest time, which includes the flow (passage) of time be added to the IGUS, and that each of these “gadgets” are dualistic. It is noted that the brain happens to have a set of veridical experiences that are congruent with the views of modern spacetime cosmology. Although some temporal experiences of the veridical system are underutilized, it is complete and all that is required for adaptation. It is also noted that all the veridical experiences have corresponding illusory experiences. As a result of natural selection, the veridical system begot the illusory system for a much better temporal system overall in order for the human to be more functional. Feynman might agree that physics successfully crossed the bridge into the cranium. Then the brain embellished it for better adaptation. With that view in mind, the compulsion to reify non-veridical experiences should be less and the two times problem might become the two times phenomenon.

## Data Availability Statement

The original contributions presented in the study are included in the article/supplementary material, further inquiries can be directed to the corresponding author/s.

## Ethics Statement

Written informed consent was obtained from the individuals for the publication of any potentially identifiable images or data included in this article.

## Author Contributions

CM and RB provided ideas for the substance of the hypothesis of this manuscript and helped edit it. RG wrote the lion’s share. All authors contributed to the article and approved the submitted version.

## Conflict of Interest

The authors declare that the research was conducted in the absence of any commercial or financial relationships that could be construed as a potential conflict of interest.

## Publisher’s Note

All claims expressed in this article are solely those of the authors and do not necessarily represent those of their affiliated organizations, or those of the publisher, the editors and the reviewers. Any product that may be evaluated in this article, or claim that may be made by its manufacturer, is not guaranteed or endorsed by the publisher.

## Footnotes

1 We are not using the completely different definition of illusion by Hoffman (2012). For him “perception has not evolved to report truth, but instead to guide adaptive behavior.” Therefore, he defines an illusion as any perceptual phenomenon that does not guide that behavior.

2 The notion that an evolutionary process e.g., natural selection, has led to an altered way we humans perceive and cope with the environment is not new. Hoffman (2012, 2019) has for several years espoused the notion that our perceptions allow us to act adaptively in the world while being ignorant of its apparent true structure. Even the spacetime of physics and actual physical objects are said not to be insights into objective reality but are species-specific adaptations that allow us to survive and reproduce.
